# Advances in the Evolutionary Mechanisms and Genomic Studies of Sexual Differentiation in Lauraceae Plants

**DOI:** 10.3390/ijms26094335

**Published:** 2025-05-02

**Authors:** Siqi Wang, Yangdong Wang, Yicun Chen, Yunxiao Zhao, Ming Gao

**Affiliations:** 1 Research Institute of Subtropical Forestry, Chinese Academy of Forestry, Hangzhou 311400, China; wsq980810@163.com (S.W.); wangyangdong@caf.ac.cn (Y.W.); yicun_chen@163.com (Y.C.); zyx_yunxiao@caf.ac.cn (Y.Z.); 2 College of Forestry, Nanjing Forestry University, Nanjing 210037, China; 3 Zhejiang Key Laboratory of Forest Genetics and Breeding, Hangzhou 311400, China

**Keywords:** Lauraceae, sexual differentiation, genome duplication, sex chromosomes, molecular breeding, ecological restoration

## Abstract

The Lauraceae family, a keystone group in subtropical evergreen broad-leaved forest ecosystems, exhibits exceptional diversity in sexual systems (including hermaphroditic flowers, functionally unisexual flowers, and pseudo-dioecy), serving as a natural model for studying plant sexual differentiation mechanisms. This review synthesizes advances in the evolutionary mechanisms and genomic studies of sexual differentiation in Lauraceae, focusing on three key areas: (1) the evolution of taxonomic classification and floral morphology, (2) molecular trajectories of sexual differentiation, and (3) challenges and future directions in sex determination research (e.g., sex-linked marker development and gene-editing-assisted breeding). Morphological and phylogenetic analyses suggest that ancestral Lauraceae species were late Cretaceous hermaphroditic trees, with recent radiation of unisexual lineages (e.g., *Cinnamomum* and *Laurus*) linked to pollinator pressure, genome duplication events (WGD), and incipient sex chromosome evolution. Despite progress, critical challenges remain, including unresolved thresholds for sex chromosome origination, unquantified molecular pathways integrating environmental signals (e.g., photoperiod, temperature) with genetic networks, and the lack of efficient sex-specific markers and genetic transformation systems. Future studies should integrate single-cell omics, epigenetic profiling, and cross-species comparative genomics to elucidate spatiotemporal dynamics and evolutionary drivers of sexual differentiation. These efforts will advance genetic improvement and ecological restoration strategies. This review provides a systematic framework for advancing plant sexual evolution theory and promoting sustainable utilization of Lauraceae resources.

## 1. Introduction

The Lauraceae family, a keystone group in subtropical evergreen broad-leaved forest ecosystems, serves as both a critical economic forest resource in southern China and a vital contributor to ecological services and industrial value. Globally, this family comprises approximately 50 genera and 2500–3000 species [1], primarily distributed in tropical regions of Southeast Asia and South America [2]. As one of the global biodiversity centers for Lauraceae, China hosts 20 genera, 423 species, and 43 varieties, with only a few deciduous taxa extending into the northern subtropical zone, while the majority are concentrated in provinces south of the Yangtze River [3].

**Figure 1:**
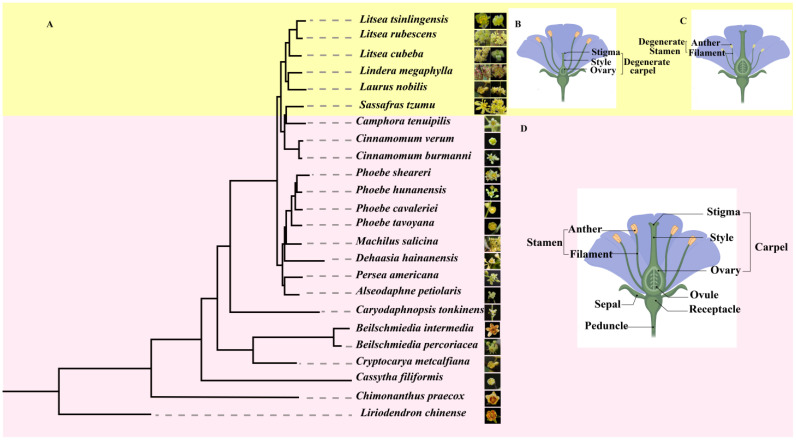
(**A**) Phylogenetic tree of representative Lauraceae species with floral morphology. The evolutionary relationships among Lauraceae species are illustrated by a maximum likelihood tree, with photographs of each species’ flowers aligned adjacent to their corresponding taxa. Color-coded branches: Yellow shading (upper clade) indicates species with bisexual flowers, while red shading (lower clade) highlights species with unisexual flowers. (**B**) Schematic diagram of a pseudo-dioecious “male” flower in Lauraceae, illustrating functional stamens and vestigial pistil. This floral structure represents pseudo-dioecious species. (**C**) Schematic diagram of a pseudo-dioecious “female” flower in Lauraceae, illustrating functional pistil and vestigial stamens. This floral structure represents pseudo-dioecious species (e.g., *Litsea cubeba*) from the Lauraceae family. (**D**) Schematic diagram of a bisexual flower in Lauraceae, illustrating functional reproductive organs. (**D**) This floral structure represents bisexual flowers (e.g., *Cinnamomum verum*, *Persea americana*) from the Lauraceae family, as depicted in the red clade of the phylogenetic tree (**A**). Key features include: functional pistil, functional stamens. Both pistil and stamens are synchronously developed, reflecting ancestral bisexual traits conserved in species of the upper clade (**A**).

The Lauraceae family exhibits remarkable diversity in sexual systems among angiosperms, encompassing hermaphroditic flowers, functionally unisexual flowers (monoecious or dioecious), and pseudo-dioecy (derived from bisexual floral primordia through selective organ abortion) [4]. Floral gender types and examples in Lauraceae are shown in Figure 1A. This unique sexual differentiation model not only serves as a natural system to dissect the genetic and developmental mechanisms underlying plant sex determination but also provides critical insights into theoretical questions such as sex chromosome evolution and male–female functional trade-offs.

The evolution of unisexual flowers represents a pivotal innovation in plant reproductive strategies. Based on developmental stages during floral ontogeny, the phenotypic features of unisexual flowers arise through four critical phases: Stage 0 (pre-initiation of sexual organ primordia), Stage 1 (early development of sexual primordia), Stage 2 (pre-meiotic phase of microspore/megaspore mother cell formation), and Stage 3 (post-meiotic phase) [5], with representative plant species for each stage summarized in Table 1. Unisexual plants can be categorized into two groups based on the timing of organ abortion: (1) True dioecious species (e.g., *Populus*), where pistil or stamen primordia are entirely suppressed at early developmental stages, resulting in strict sexual dimorphism [6]. (2) Pseudo-dioecious species, whose floral primordia initially retain bisexual potential but later undergo spatiotemporally selective abortion of specific reproductive organs. The latter group further divides into three subtypes (Table 1): Type I: Both male and female flowers retain vestigial organs (e.g., *Silene conoidea* [7], *Vitis vinifera* [8], *Litsea cubeba* (Lour.) Pers [9]). Type II: Male flowers retain rudimentary pistil primordia, while female flowers lack stamen primordia (e.g., *Carica papaya* [10]).Type III: Female flowers retain vestigial stamen primordia, while male flowers lack pistil primordia (e.g., *Vernicia fordii* [11], *Rumex acetosa* [12], *Coccinia grandis* [13]). This diversity highlights that the evolution of unisexual flowers is mediated by multiple pathways—including primordium suppression, programmed organ abortion, and epigenetic regulation—refining nature’s selective optimization of reproductive resource allocation.

Research on the mechanisms of sexual differentiation in pseudo-dioecious Lauraceae species remains significantly understudied. Despite morphological similarity between male and female individuals before reproductive maturity and their prolonged juvenile phases (typically >5 years), molecular studies have identified sex-biased gene expression divergence and epigenetic marker differences [10]. However, existing research predominantly focuses on morphological descriptions and cytological observations, with limited systematic exploration of key regulatory genes, hormone interaction networks, and environmental response mechanisms. Furthermore, debates persist regarding the presence, degeneration level, and evolutionary relationships of sex chromosomes in Lauraceae with those of closely related taxa (e.g., *Magnoliales*) [11,12].

This review synthesizes recent advances in molecular biology and evolutionary developmental biology, emphasizing three critical areas: (1) the evolution of Lauraceae classification systems and floral morphological adaptations, (2) molecular trajectories of sexual differentiation, and (3) current challenges and future directions in sex determination research (e.g., sex-linked marker development and gene-editing-assisted breeding). By systematically integrating these insights, we aim to unravel the unique and universal principles underlying sexual differentiation in Lauraceae, offering novel perspectives for forest genetic improvement and advancing plant sex evolution theory.

## 2. Sex Determination Mechanisms in Angiosperms

The sex determination of angiosperms is a multi-level regulatory process that begins with the formation of floral meristems and is completed through the coordinated actions of sex-determination genes, sex chromosomes, hormone signaling, and epigenetic modifications. Its core mechanisms can be summarized into three major modules.

### 2.1. Sex Chromosome Systems: Evolution from Autosomes to Sex-Determining Factors

Plant sex chromosomes are mostly derived from autosomes, typically originating from ancestral chromosomes carrying sex-related mutations. Plant sex chromosome systems are primarily classified into three categories. (1) ZW System: Females are heterogametic (ZW), and males are homogametic (ZZ), as seen in the willow family (Salicaceae, e.g., *Populus* spp.). (2) XY System: Males are heterogametic (XY), and females are homogametic (XX), such as in common sorrel (*Rumex acetosa*). (3) UV System: Females have a single U chromosome, and males have a single V chromosome, commonly found in bryophytes [13,14].

The functions of sex chromosomes are complex:

(1) Dosage-dependent sex determination: In Rumex and Humulus, sex is determined by the ratio of X chromosomes to autosomes (X/A). For example, a female phenotype arises when the X/A ratio ≥ 1, a male phenotype when ≤0.5, and intersex flowers develop at intermediate ratios [11].
(2) Heterochromatinization and recombination suppression: The Y chromosome of *Carica papaya* exhibits five heterochromatic knobs, with its non-recombining regions harboring sex-determining genes SR and SVP-like, which govern hermaphroditic (Y^h^ chromosome) and male (Y chromosome) phenotypes [15,16].
(3) Studies in *Silene latifolia* and *Phoenix dactylifera* reveal a positive correlation between sex chromosome heterochromatization and recombination suppression, supporting the “degeneration–differentiation” model of sex chromosome evolution. According to this model, sex chromosomes originate from a pair of autosomes, with differentiation triggered by the emergence of a sex-determining gene. Chromosomal inversions near this gene suppress recombination, leading to progressive gene loss and accumulation of repetitive sequences. Heterochromatization, marked by transposon proliferation and stabilized by DNA methylation and other epigenetic modifications, reinforces recombination suppression, ultimately resulting in heteromorphic sex chromosomes (e.g., XY/ZW systems) [17].

### 2.2. Sex-Determining Genes: From Single-Gene Regulation to Multifactor Interaction Networks

#### 2.2.1. Strict Dioecy Systems

In *Populus*, sex determination relies on interactions between Y chromosome-specific genes. The Y-linked gene *FERR-R* produces siRNA that induces methylation of its own promoter and degrades *FERR* mRNA, suppressing pistil primordium development [6]. However, recent studies suggest *ARR17* may be the core sex-determining gene in poplar [18]. Its Y-specific inverted repeats regulate stamen development via small RNAs, though this remains debated. While *FERR-R* and *ARR17* both play roles, their hierarchical relationship is unclear. Some propose *ARR17* acts as a “sex switch” (female development when active, male when silenced), whereas *FERR-R* primarily suppresses female traits through epigenetic silencing.

In *Hippophae rhamnoides* ssp. *sinensis*, MADS-box genes exhibit sex-specific expression patterns. B-class genes (*HrMADS55*, *78*, *42*) are highly expressed in stamens, and *HrMADS62* in male flower bracts, potentially determining male organ identity. D-class gene *HrMADS69* shows high expression in pistils, likely regulating female organ development [19].

#### 2.2.2. Pseudo-Dioecy Systems

In pseudo-dioecious plants, sex differentiation exhibits developmental stage specificity, with key regulatory genes acting at distinct phases:

Pre-primordia initiation regulation: In spinach (*Spinacia oleracea*), B-class genes *SpPI* and *SpAP3* determine sex by controlling stamen primordium formation. Pan-genome analyses reveal associations between Y chromosome degeneration and structural variations (pan-SV) [14].

Early primordia regulation: In kiwifruit (*Actinidia* spp.), the Y chromosome genes *SyGI* (a cytokinin-responsive regulator) and *FrBy* (a stamen development maintenance factor) form a dual-factor model, suppressing pistil and stamen development, respectively [20]. In melon (*Cucumis melo*), *CmWIP1* promotes male flower formation by inhibiting the ethylene biosynthesis gene *CmACS7* [16].

Post-meiotic regulation: In persimmon (*Diospyros kaki*), the Y-linked gene *OGI* encodes a small RNA that silences the autosomal gene MeGI (a homeodomain transcription factor), thereby releasing its suppression of stamen development and establishing sexual dimorphism [6].

### 2.3. Epigenetic Regulation: A Multi-Layered Network Integrating Transposon Dynamics

Sexual differentiation in plants is dynamically regulated by three core epigenetic mechanisms—DNA methylation, histone modifications, and non-coding RNA networks—which collectively mediate environmental-genome crosstalk.

#### 2.3.1. DNA Methylation Synergizes with Metabolic–Hormonal Signaling

By integrating whole-genome bisulfite sequencing (WGBS) and transcriptomic analyses, Hu et al. elucidated the dual regulatory role of S-adenosylmethionine synthase (SAMS) in *Arabidopsis*: SAMS-mediated dynamic DNA methylation (notably reduced CG-site methylation) synergizes with ethylene signaling to disrupt the expression of ABCE floral organ identity genes, resulting in SAM-overexpressing (SAMOE) transgenic plants with abnormal petal/sepal numbers, stamen petaloidy, and pistil developmental defects [21]. This metabolite–epigenetic–hormone crosstalk is further corroborated in papaya (*Carica papaya*), where CHH-type methylation differences in the promoter of CpHUA1 correlate with transitions among three sex types (male, female, and hermaphrodite), suggesting that methylation reprogramming may lock sex developmental trajectories through epigenetic memory [22].

#### 2.3.2. Spatiotemporal Specificity of Histone Modifications

Research on melon unisexual flower development deciphers the spatiotemporal logic of histone codes: Through ChIP-seq and spatial profiling of H3K27me3 (repressive mark) and H3K9ac (activating mark) across five organs in wild-type and mutant plants, sex-specific histone modifications were found to dynamically regulate ethylene-responsive genes and MADS-box family genes (e.g., *CmACS7* and *CmWIP1*). For instance, H3K9ac enrichment at ethylene biosynthesis loci in stamen primordia activates male programs, while H3K27me3 deposition in pistil primordia suppresses male pathways via chromatin compaction. The spatiotemporal decoding of this “histone modification code” provides an epigenetic blueprint for unisexual flower development [20].

#### 2.3.3. Non-Coding RNAs Drive the Evolution of Sex Determination Systems

Studies on *Diospyros* spp. (persimmon) provide evolutionary insights into epigenetic regulation: Comparative genomic and transcriptomic screening identified 22 candidate genes, ultimately pinpointing the Y chromosome-encoded small RNA OGI. Phylogenetic and functional analyses revealed that OGI silences the autosomal female-suppressing gene MeGI via RNA interference, establishing a “Y-linked epigenetic switch–autosomal target” axis. This model not only deciphers the genetic basis of dioecy but also highlights the critical role of epigenetic elements in sex chromosome evolution.

## 3. Floral Development and Morphological Diversity in Lauraceae

As a pivotal lineage within the magnoliids, the Lauraceae family exhibits floral morphology and developmental mechanisms that demonstrate a remarkable balance of conservatism and adaptability, establishing it as an ideal model system for studying plant evolution and sexual differentiation. This review explores these aspects through multiple dimensions: taxonomic systems, morphological traits, evolutionary trajectories, and ecological drivers.

### 3.1. Evolution of Lauraceae Classification Systems and Floral MorphologicalMechanisms

#### 3.1.1. Classic Classification Systems and Morphological Basis

The classification of Lauraceae. In the period of Carl von Linné (1753), there were only two genera, Laurus and Cassytha [23]. In 1957, Kostermans proposed a classification system for the Lauraceae family, primarily based on morphological traits such as inflorescence characteristics and ovary position. This system divided Lauraceae into two subfamilies (Cinnamomoideae and Cassythoideae) and six tribes, including Litseeae, Perseeae, and Cinnamomeae, encompassing 66 genera such as *Neocinnamomum*, *Machilus*, and *Cinnamomum*. Kostermans’ classification emphasized the distinct subfamilial status of the parasitic vine genus *Cassytha* (Cassythoideae) [24]. However, its limitations lay in an over-reliance on morphological features, and insufficient consideration of wood anatomical and molecular evidence, leading to disputes over tribal placements of certain genera (e.g., *Dehaasia* vs. *Cryptocarya*).

In 1996, van der Werff and Richter introduced a revised system integrating wood anatomical features (e.g., vessel perforation types, ray tissue heterogeneity) and inflorescence morphology. They redefined the tribes *Machileae*, *Perseeae*, and *Cryptocaryeae*, reclassified *Cinnamomum* and *Phoebe* into *Perseeae*, and adjusted the systematic position of *Sassafras* based on oil cell distribution patterns. While innovative in incorporating micro-anatomical evidence, this system still faced limitations: molecular phylogenetic studies revealed that some tribes (e.g., *Perseeae*) were non-monophyletic, and its treatment of Asian-endemic genera (e.g., *Neocinnamomum*) failed to reflect true evolutionary relationships, highlighting discrepancies between morphological traits and molecular data [25].

Neither system resolved key evolutionary nodes in Lauraceae, such as the ambiguous generic boundaries between *Machilus* and *Phoebe*.

#### 3.1.2. Molecular Phylogenetic Reconstruction of Taxonomic Frameworks

**Figure 2:**
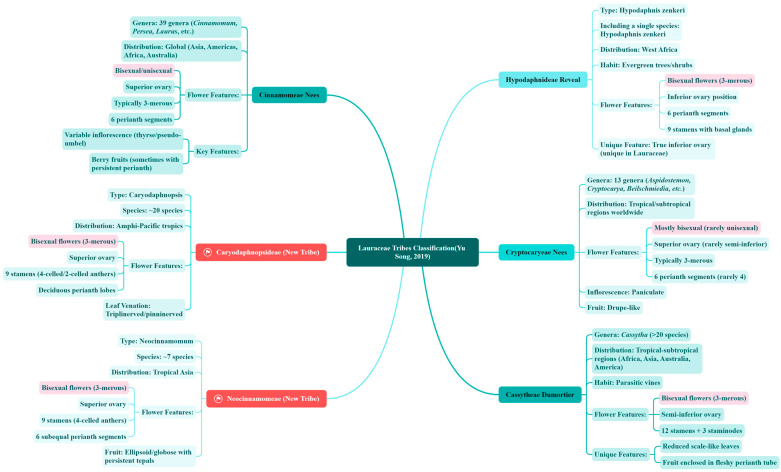
The classification framework of six tribes of Lauraceae based on molecular systematics [26].

With the application of genomics technology, the classification system of Lauraceae has undergone significant adjustments. Recent integrative analyses of plastid genomes, nuclear genomes, and transcriptomic data have significantly refined Lauraceae classification. A research team from the Xishuangbanna Tropical Botanical Garden (Chinese Academy of Sciences) reconstructed a phylogenomic framework using plastid genomes of 131 species, resolving six tribes and nine clades, including *Cassytheae*, *Cryptocaryeae*, and *Laureae*. The *Laureae* tribe was further divided into four monophyletic subclades (e.g., *Cinnamomum–Sassafras* clade, *Laurus–Neolitsea* clade), with the proportion of hermaphroditic flowers aligning with molecular phylogenetic results [26]. The core information of each tribe, including genera, species, distribution, habits, floral characteristics, fruit characteristics, etc., is summarized in Figure 2.

Phylogenetic Controversies in the Systematics of *Cassytha*: Genomic studies of *Litsea cubeba* reveal conflicting phylogenetic signals regarding the placement of *Cassytha*. Nuclear gene trees position *Cassytha* as sister to other Lauraceae lineages, while plastid phylogenies nest it within the family, suggesting potential incomplete lineage sorting (ILS) or ancient hybridization events [12]. Future research should employ 3D genomics and phenomics to resolve phylogenetic conflicts in Cassytha. Additionally, nuclear genomic data indicate a close sister relationship between *Litsea* and *Cinnamomum* (traditionally classified into distinct tribes), challenging conventional tribal boundaries and underscoring the need for redefining higher-level taxonomy using multi-omics datasets [12]. Mitochondrial phylogenies further highlight stable topological relationships between the *Cryptocaryeae* and *Laureae* tribes, yet conflicts arise in the placement of certain *Caryodaphnopsis* species compared to nuclear gene trees, emphasizing the necessity of integrating multi-omics data [27].

#### 3.1.3. Whole-Genome Duplication (WGD) Events in Lauraceae

Lauraceae has undergone two WGD events: the first occurred prior to the divergence of Laurales and Magnoliales [15], and the second coincided with the early radiation of Lauraceae. These WGD events facilitated the expansion of the monoterpene synthase (mono-TPS) gene family, driving the diversification of essential oil compounds (e.g., citral, linalool) characteristic of Lauraceae species [12].

The inflorescence types in Lauraceae encompass spicate, racemose, paniculate, and umbellate forms, with their evolutionary trajectory following a progressive transition: spicate → spicate-paniculate → cymose-paniculate → umbellate [16]. Molecular phylogenetic studies reveal that the *FUWA* gene drives inflorescence morphological shifts by regulating meristem activity, leading to shortened inflorescence axes and compressed lateral floral organs, serving as the genetic basis for these transitions. Transcriptomic analyses indicate that unisexual flower formation correlates with functional divergence of the *AGL6* gene, and unisexual lineages generally exhibit lower inflorescence complexity compared to hermaphroditic species. Pollination ecology research demonstrates that the compact structure of umbellate inflorescences significantly enhances pollination efficiency.

This adaptive evolution is closely linked to the whole-genome duplication (WGD) events in Lauraceae, which accelerated inflorescence innovation through genetic network reorganization [12].

#### 3.1.4. Conservatism and Diversity of Floral Organs in Lauraceae

The conservatism of Lauraceae floral organs is exemplified by their pan-familial structural stability: two whorls of six (rarely four) tepals, with the outer whorl often sepaloid (e.g., *Cryptocarya*) and fused into a cup-shaped hypanthium; four whorls of stamens (innermost reduced to staminodes) with glandular filaments secreting methyleugenol as specialized chemical signals; and a consistent monocarpellate ovary, single ovule, and discoid stigma (e.g., trilobed stigma in *Cinnamomum*) [28]. Diversity manifests in tepal differentiation (e.g., sepaloid specialization in *Cryptocarya* vs. petal retention in *Litsea* for pollination adaptation), seven pollen morphological types (including *Laurus* with spine-based protrusions mechanically interlocking beetle surfaces), and anther dehiscence mechanisms (e.g., lateral slit orientation in *Persea* to enhance pollen release) [29].

Genomic evidence reveals that the conserved floral organ traits in Lauraceae are rooted in deeply conserved molecular regulatory mechanisms. Whole-genome analysis of *Cinnamomum camphora* demonstrates broad-spectrum expression of ABCDE model homologs: *AGL6* is highly expressed in tepals, *AP3/PI* homologs show significant expression in both tepals and stamens, while *AG/STK* genes are specifically expressed in pistils and stamens. This expression pattern aligns with ancestral features observed in basal angiosperms (e.g., *Nymphaea*) [16], indicating that Lauraceae retains the primitive angiosperm trait of broad activation of floral organ identity genes, resulting in undifferentiated tepals rather than distinct sepals and petals.

Comparative studies of the MADS-box gene family reveal that Lauraceae species possess a significantly higher number of MIKC genes (53) compared to *Amborella trichopoda*, yet fewer than other closely related Lauraceae lineages. Notably, subfamilies critical for floral development—such as *SEP*, *AGL6*, and *SVP*—have undergone expansion in Lauraceae, while the monocot-specific *OsMADS32* gene is entirely absent. This differential evolution of gene families likely accounts for the pronounced divergence in floral organ morphology between Lauraceae and *Amborella*, supporting molecular phylogenetic evidence for the reclassification of *Amborella* outside the Laurales order.

Future research should employ 3D genomics and phenomics to resolve phylogenetic conflicts in *Cassytha* and decode the genetic regulatory networks underlying monoterpene metabolism.

### 3.2. Sexual Differentiation and Evolutionary Trajectories in Lauraceae

#### 3.2.1. Ancestral Reconstruction and Phylogeny

**Figure 3:**
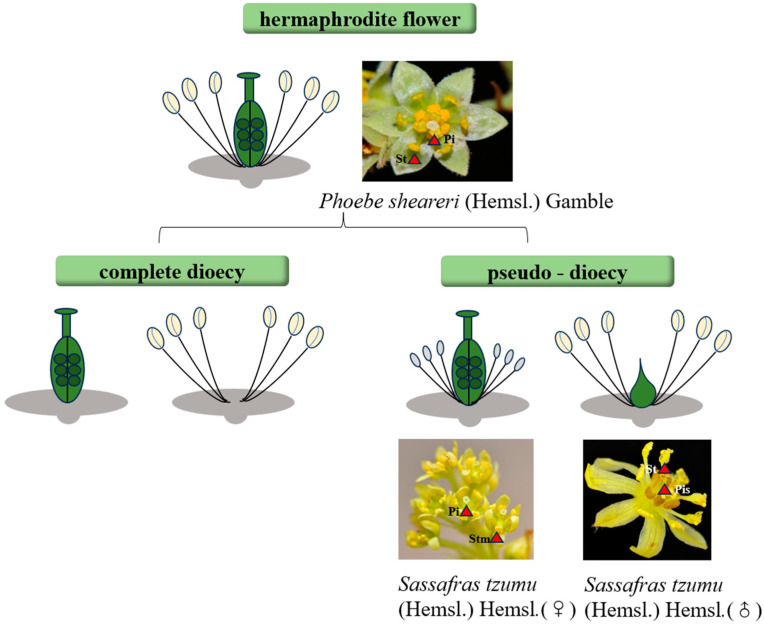
Floral diversity and molecular mechanisms. Floral gender types and examples in Lauraceae. Hermaphrodite flower: represented by *Phoebe sheareri* (Hemsl.), Pseudodioecy: illustrated by *Sassafras tzumu*. Pi: Pistil, Pis: Pistillode, St: Stamen, Stm: Degenerate stamen.

Molecular clock and fossil evidence indicate that the ancestral Lauraceae species were late Cretaceous hermaphroditic trees with radially symmetrical flowers. Unisexual lineages (e.g., *Cinnamomum*, *Laurus*) rapidly diversified during the Eocene [13]. The evolutionary tree of representative Lauraceae species is shown in Figure 1A (This figure is reprinted from, Figure 3); male, female, and hermaphroditic floral diagrams are illustrated in Figure 1B–D.

#### 3.2.2. Transitional Lineages and Evolutionary “Intermediate States”

Unisexual-to-Hermaphroditic Transitions: Comparative transcriptomics has identified key regulatory genes (e.g., *AGAMOUS* homologs) whose expression patterns directly correlate with floral organ development [16].

Convergent Evolution of Unisexuality: Chen et al. reconstructed a Lauraceae phylogeny using 275 single-copy genes from transcriptomes of 22 species across 15 genera, revealing convergent evolution of unisexual flowers.

Mixed Sexual Systems: The genus *Machilus* includes both hermaphroditic and unisexual species, possibly reflecting transitional stages of sexual differentiation [12]. Sexual types of representative Lauraceae species are summarized in Figure 2.

#### 3.2.3. Genomic Insights into Sex Determination Mechanisms

Genome sequencing of Lauraceae species has achieved significant progress, with 14 genome assemblies currently completed across nine species, including *Cinnamomum camphora* and *Litsea cubeba* (Table 2).

The family Lauraceae ranks fourth in species richness among arborescent groups within the class Magnoliopsida [38], comprising approximately 50 genera and 2500–3000 extant species globally [39]. However, genomic research on Lauraceae significantly lags behind other economically important plant groups. As of 2025, the NCBI genome database contains only nine fully sequenced Lauraceae genomes. Current studies predominantly focus on secondary metabolite biosynthesis pathways. Notably, molecular mechanisms of sexual differentiation have been preliminarily explored only in *Litsea cubeba*, while critical areas like epigenetic regulation (e.g., dynamic DNA methylation modifications) and non-coding RNA interaction networks remain understudied, lacking systematic frameworks.

In *Litsea cubeba*, the salicylic acid (SA)-responsive factor *LcTGA10* has been confirmed to regulate SA metabolism and participate in the early abortion of stamens in female flowers. Specifically, SA levels in female flowers peak before stamen abortion (2.7 times higher than in male flowers) [7]. Overexpression of *LcTGA10* induces pistil degeneration in male flowers, while silencing this gene leads to abnormal development of hermaphroditic flowers [40]. Additionally, the hypothetical protein Lcu01G_02292, which shows differential expression between unisexual and bisexual flowers, is hypothesized to play a role in sex determination [12]. Notably, SOC1-like genes are expanded in both *L. cubeba* (seven members of SOC1) and C. kanehirae (eight members of SOC1) [18]. Consistent with the expanded SOC1 clade, the SVP clade is also expanded, and it counts five members in *L. cubeba*. It has been reported that the interaction of SOC1 and AGL24 from the SVP clade integrates flowering signals in *Arabidopsis* [41]. Both the expanded SOC1 and SVP clades could be involved in complex flowering regulation networks and could relate to differential regulation of dioecious plant flowering [12].

However, the specific roles of TGA10, SOC1, and SVP clades in sex differentiation of *Litsea cubeba* remain unknown.

#### 3.2.4. Environmental Factors Drive Gender Differentiation in Lauraceae

In Lauraceae plants, the gender differentiation strategy of *Lindera glauca* exemplifies a strong correlation between external environmental factors and the evolution of the reproductive system. The mainland populations of *Lindera glauca* ensure reproductive assurance through a mixed strategy of dioecious sexual reproduction and apomixis. In contrast, the Japanese island populations rely solely on apomixis. Paleo-niche models and population genomics analyses indicate that the Japanese lineage became isolated from its mainland ancestors after undergoing a severe population bottleneck during the Mid-Pleistocene (around 0.3 Ma). Habitat fragmentation during the Last Glacial Maximum (LGM) led to the extinction of male plants, and apomixis emerged as an adaptive strategy for the island populations to cope with pollinator scarcity [42].

Conversely, the mainland populations maintained genetic diversity throughout the glacial–interglacial cycles. Their mixed reproductive strategy allows for the maintenance of genetic variation via sexual reproduction and overcomes pollination limitations in low-density populations through apomixis [42]. This case unveils the mechanism by which historical climate change drives the differentiation of reproductive strategies by shaping population dynamics, such as bottleneck effects and founder effects. It also validates the adaptive expansion of Baker’s Law in woody plants [43]. That is, when the sexual system is confronted with extreme environmental pressures, apomixis may be retained by natural selection as a “reproductive insurance” (Self-compatibility and establishment after “long-distance” dispersal). Moreover, this case reveals how population history influenced by paleoclimate change, including bottlenecks and expansions, selects specific gender differentiation strategies, providing an ecological perspective for understanding the evolution of gender systems in Lauraceae plants.

#### 3.2.5. Current Challenges in Lauraceae Sex Determination Research

The study of sex determination mechanisms in Lauraceae faces multiple bottlenecks:

Immature Genetic Transformation Systems: The lack of efficient genetic transformation protocols severely hampers functional validation of candidate genes.

Genomic Complexity and Functional Redundancy: Repeated whole-genome duplication (WGD) events have generated multiple paralogs of key genes (e.g., *AG*, *ARR12*), complicating mechanistic studies. Most species lack well-defined sex chromosomes, making it difficult to delineate sex determination regions (SDRs).

Spatiotemporal resolution of epigenetic regulation: Current technologies struggle to simultaneously track DNA methylation dynamics (e.g., CG island shifts in the *CmWIP1* promoter), histone modifications (e.g., H3K27ac), and non-coding RNA networks at single-cell resolution. Additionally, the prolonged growth cycles of Lauraceae species (e.g., 3–5 years for *Persea americana* to flower) hinder cross-generational validation of epigenetic effects.

Ambiguous Environment–Gene Interactions: Quantitative models are lacking to explain how photoperiod and temperature signals regulate sexual differentiation via epigenetic modifications or hormone pathways. Laboratory simulations also fail to replicate the complex feedback inherent in natural pollinator networks.

## 4. Perspectives: New Frontiers in Multi-Omics Integration and Evolutionary Developmental Research

With advancements in genomics, transcriptomics, proteomics, and metabolomics, research on sexual differentiation in Lauraceae is entering an era of multi-dimensional integration. Future studies should prioritize the following directions:

### 4.1. Multi-Omics-Driven Dissection of Sex Determination Networks

Research on the mechanisms of plant sex determination and differentiation based on multi-omics data has been widely carried out across multiple taxonomic groups, yielding systematic achievements. For example, Wan Yinglang’s team completed de novo assembly of the *Areca catechu* (betel nut) genome and integrated chromatin accessibility sequencing (ATAC-seq) with transcriptomic analysis to reveal that *Areca catechu* regulates bisexual flower development through epigenetic modulation of the jasmonic acid (JA) biosynthesis pathway. The core mechanism involves regulating the expression of B-class transcription factors in the floral development ABC model to determine the differentiation direction of female and male flowers [44].

Additionally, multi-omics studies on *Salix babylonica* (weeping willow) have deciphered its sex-determination mechanism: In male individuals (15ZZ), two Z chromosomes carry incomplete *ARR17-like* sequences that completely suppress the intact *ARR17-like* genes (female-determining factors) on four autosomes of chromosome 19, leading to male determination. In female individuals (15ZW), the incomplete sequence on one Z chromosome fails to suppress the intact genes on autosomes, allowing female-determining factors to be expressed and enabling stable female sex determination. This discovery, through integrating genomic and transcriptomic data, clearly illustrates the interplay between sex chromosome sequence variations and autosomal gene expression [45].

Currently, high-quality chromosome-level genomes of multiple Lauraceae species (e.g., *Litsea cubeba*, *Cinnamomum kanehirae*) have been assembled. Drawing on the strategy of “whole-genome assembly + ATAC-seq” used in *Areca catechu* research, these Lauraceae genomes can precisely map sex-related genomic regions and identify key candidate genes and their regulatory elements. For example, in pseudo-dioecious species like *Sassafras tzumu*, comparative genomic and transcriptomic analyses between female and male plants can reveal how structural variations (e.g., deletions, duplications) on sex chromosomes or autosomes lead to stamen/pistil degeneration or functional differentiation.

Inspired by the “genome + transcriptome” approach used in *Salix babylonica* to dissect sex chromosome-autosome interactions, single-cell transcriptomics in Lauraceae can capture rare cell subpopulations during the early stages of floral primordium development (e.g., critical nodes in the transition from bisexual to unisexual flowers), identifying key transcriptional modules associated with sex determination. For instance, during sex differentiation in *Litsea cubeba*, single-cell data can characterize interaction networks between hormone-responsive cells (e.g., cells enriched in cytokinin/auxin signaling) and floral organ-specifying cells (e.g., stamen primordium, carpel primordium cells), revealing how MADS-box genes (e.g., *AG*, *SEP*) and hormone pathway genes (e.g., *ARR12*, *AUX/IAA*) coordinately regulate sex differentiation.

### 4.2. Spatiotemporal Specificity of Epigenetic Regulation

In Lauraceae, researchers can draw on the integrated strategy of WGBS and RNA-seq used in *Arabidopsis* to analyze whether SAMS homologous genes in species such as avocado (*Persea americana*) and camphor tree (*Cinnamomum camphora*) affect the expression patterns of ethylene synthesis genes (e.g., CYP703 homologs) or ABCE floral organ identity genes (e.g., CmWIP1) through CG/CHH methylation, and validate the conservation of the “metabolism–epigenetics–hormone” regulatory axis during the differentiation of unisexual flower primordia (e.g., *Litsea cubeba*). By referring to the non-coding RNA screening approach in *Diospyros*, comparative genomics can be used to mine lncRNAs/miRNAs with sex-differential expression in Lauraceae, exploring whether a regulatory axis similar to “OGI-MeGI” exists—where non-coding RNAs drive unisexual flower formation by targeting and silencing or activating key genes such as the MADS-box family. Leveraging the ChIP-seq and laser capture microdissection techniques used in melon, researchers can map the enrichment of H3K9ac (an activation mark) in the promoter regions of stamen development genes (e.g., AcAMS) and the deposition of H3K27me3 (a repression mark) in female-inhibiting genes within the carpel and stamen primordia of *Litsea cubeba* and other species, combining single-cell epigenetic analysis to decode the epigenetic heterogeneity in sexual primordia.

### 4.3. Environment–Gene Interactions and Ecological Adaptation

In the plant kingdom, the phenomenon of environmental signals (such as photoperiod, temperature, soil microbiome) regulating sex differentiation through epigenetic mechanisms, hormonal signaling, and ecological interactions is widespread. For example, in *Cucumis sativus* (cucumber), under conditions of high temperature and long-day light, changes in the methylation level of the ethylene synthesis gene CsACO3 directly inhibit pistil development, and this process involves cross-regulation with the cytokinin signaling pathway [46]. Treatment of *Phellodendron amurense* with 5-azacytidine leads to DNA demethylation, transforming male plants into functional female plants, and this sex reversal is heritable [47]. Male and female plants of the genus *Populus* differentially recruit rhizosphere microorganisms by releasing sex-specific phenolic compounds, thus regulating the population sex ratio [48]. However, there are no relevant reports on environmental factors in the study of sex differentiation in plants of the Lauraceae family.

In later research, by simulating multi-environmental systems (photoperiod, temperature, soil microbiome) combined with integrated genomic–epigenomic analysis, the ecological signaling pathways of sex differentiation in Lauraceae plants will be revealed. It will be determined whether the DNA methylation variations induced by habitat fragmentation are heritable, and thus affect the population sex ratio. Applying metagenomics to analyze the co-evolutionary relationship between the volatile metabolites of Lauraceae plants and pollinators (such as beetles and bees) will clarify the feedback mechanism between sex differentiation and ecological interactions.

### 4.4. Comparative Genomics and Evolutionary Reconstruction

Leveraging Lauraceae’s diverse sexual systems (hermaphroditic, unisexual, and pseudo-dioecious flowers), cross-species comparative studies will track duplication-functional divergence events in B-class (*AP3/PI*) and C-class (*AG*) genes, revealing their shift from floral organ development to sex determination. Phylogenomic analyses will test whether recombination suppression regions in Lauraceae follow the ‘two-step model’—a paradigm exemplified by Silene species where recombination suppression expands progressively following the accumulation of deleterious mutations on proto-Y chromosomes (e.g., SlX1/SlY1 with dS = 0.21 in S. latifolia). In contrast, Salicaceae’s XY/ZW systems challenge this model: their sex-determining regions (SDRs) undergo rapid turnovers via transposon-mediated genomic rearrangements (e.g., Helitron-driven relocation of RR genes in *Populus*), and siRNA-induced epigenetic silencing (e.g., ARR17-like paralogs in Salix) [49]. Integrating fossil pollen data (e.g., *Lauraceaephyllum*) and molecular clock models will reconstruct paleoenvironmental contexts of key sex differentiation events since the Cretaceous, predicting adaptive responses to climate change and providing multi-dimensional insights into Lauraceae’s evolutionary mechanisms [50].

### 4.5. Application-Oriented Molecular Breeding

Exploiting key sex-determination genes in Lauraceae, early sex identification markers (e.g., *AP3*-based male-specific promoters or *OGI*-homologous sRNAs) can enable precision breeding. CRISPR-Cas9-mediated knockout of *ARR12* or overexpression of *AG* may generate high-yield unisexual female lines, boosting fruit production in economically vital species (e.g., avocado, *Persea americana*). Additionally, modulating methylation levels of *CmWIP1* homologs could optimize dioecious population designs for ecological restoration. These integrated technologies will bridge basic research and applied precision breeding, advancing Lauraceae’s role in sustainable forestry and ecosystem engineering.

Research on sexual differentiation in Lauraceae is transitioning from morphological characterization to mechanistic dissection and artificial regulation. Integrating multi-omics approaches, epigenetic decoding, and environmental interaction analyses holds promise for uncovering universal principles of sex determination in magnoliids and lineage-specific mechanisms in Lauraceae. These advancements will not only enrich evolutionary developmental biology (evo-devo) theory but also deliver innovative solutions for forestry production, ecological restoration, and climate change resilience.

## Figures and Tables

**Table 1:** Regulatory patterns of unisexual flowers.

Degenerative Stage	Species	Patterns of Control	References
Stage 0	*Spinacia oleracea*	XY system with Y-linked insertion duplications suppressing recombination	[1,2]
Stage 1	*Cucumis melo*	Regulation of ethylene synthesis pathway and epigenetic regulation (DNA methylation); The key factor in sex determination: CRC	[3,4]
Stage 1	*Actinidia* spp.	The SyGI gene inhibits carpel development. The FrBy acts for the maintenance of male functions	[5,6]
Stage2	*Litsea cubeba*	Hormonal regulation	[7]
Stage 3	*Diospyros lotus*	Epigenetic regulation (Srna)	[8]
Stage 3	*Asparagus officinalis*	The two-mutation model	[9]

**Table 2:** The chromosomal-level genomic information of sequenced Lauraceae species.

Number	Species	Genus/Tribe	Genome Size (Mb)	Chromosome Number (2n)	Sequencing Technology	Assembly Level	Research Focus	References
1	*Litsea cubeba*	*Laureae*	1325.7	24	PacBio CLR + Hi-C	Chromosome	Association between Monoterpene Synthesis and Sex Evolution	[16]
2	*Litsea coreana*	*Laureae*	1139.5	24	Illumina + PacBio CCS + Hi-C	Chromosome	Flavonoid Metabolism and Stress Resistance Mechanisms	[15]
3	*Lindera glauca*	*Laureae*	2092.2	24	Illumina + Nanopore + Hi-C	Chromosome	Parthenogenesis and Heterozygous Genomic Features	[30]
4	*Cinnamomum kanehirae*	*Cinn amomeae*	730.7	24	Illumina + PacBio CLR + Chicago + Hi-C	Chromosome	Terpenoids and Fatty Acid Biosynthesis Pathways	[18]
5	*Cinnamomum camphora*	*Cinn amomeae*	755.4	24	PacBio CCS + Hi-C	Chromosome	Molecular Basis of Chemotypic Diversity in Terpenoids	[19]
6	*Cinnamomum camphora*	*Cinn amomeae*	723.1	24	Illumina + PacBio CCS + Hi-C	Chromosome	Phylogenetics and Key Genes in Essential Oil Biosynthesis	[31]
7	*Cinnamomum camphora*	*Cinn amomeae*	719.9	24	Illumina + PacBio CCS + Hi-C	Chromosome	Genome Resequencing and Chemotype Evolution	[11]
8	*Cinnamomum camphora*	*Cinn amomeae*	785.0	24	PacBio CCS + Hi-C		Evolution and Terpenoid Biosynthesis	[32]
9	*Cinnamomum burmanni*	*Cinn amomeae*	1177.6	24	Illumina + PacBio CLR + Hi-C	Chromosome	Terpenoid Synthesis and Mining of Disease-Resistance Genes	[33]
10	*Phoebe bournei*	*Perseeae*	989.2	24	PacBio CLR	Scaffold	Wood Properties and Secondary Metabolic Pathways	[34]
11	*Phoebe bournei*	*Perseeae*	941.8	24	PacBio CLR; BioNano and Hi-C	Chromosome	Terpenoid biosynthesis, WGD evolutionary mechanisms, and disease resistance applications	[15]
12	*Persea americana*	*Perseeae*	912.6	24	PacBio CLR	Chromosome	Paleopolyploidization and the Origin of Sex Chromosomes	[35]
13	*Persea america-na*	*Perseeae*	913.0	24	Illumina and PacBio CCS	Chromosome	Evolutionary context, metabolic pathways, and fruit traits	[36]
14	*Cinnamomum chago*	*Cinn amomeae*	785.0	24	PacBio CCS + Hi-C	Chromosome	Conservation of Endangered Resources and Terpenoid Synthesis	[37]

## Data Availability

The data used in this review all come from published academic literatures. All the cited literatures have been completely listed in the reference list. The data in these literatures can be obtained through the official websites of the journals where they are published. For most journals, readers need to access them through the subscription services of their affiliated institutions or by paying a fee. If readers are unable to obtain the data in certain literatures through conventional channels, they can apply for data sharing from the original authors in accordance with the relevant regulations of each journal. We have made every effort to ensure the accuracy and traceability of the cited data in the original literatures.
